# Molecular profiling of nematode associates with *Rhynchophorus ferrugineus* in southern Italy

**DOI:** 10.1002/ece3.5865

**Published:** 2019-11-28

**Authors:** Francesca De Luca, Elena Fanelli, Monica Oreste, Gianluca Scarcia, Alberto Troccoli, Alessio Vovlas, Nicola Trisciuzzi, Eustachio Tarasco

**Affiliations:** ^1^ Institute for Sustainable Plant Protection (IPSP)‐CNR Bari Italy; ^2^ Section of Entomology and Zoology Department of Soil, Plant and Food Sciences University of Bari “A. Moro” Bari Italy; ^3^ A. P. S. Polyxena Conversano Italy; ^4^ Centro Ricerca Sperimentazione e Formazione in Agricoltura (CRSFA) Locorotondo Italy

**Keywords:** molecular biodiversity, nematode fauna, red palm weevil, reverse taxonomy

## Abstract

A survey of nematodes associated with the red palm weevil *Rhynchophorus ferrugineus* was conducted in southern Italy in 2015 and 2016 in order to create a species inventory and obtain data about nematode biodiversity. A total of 70 insect samples (pupae and adults) were collected from infested *Phoenix canariensis*, *Phoenix dactylifera*, and *Chamaerops humilis* palms in three Italian Regions: sampling took place at 11 locations in Apulia, 1 in Basilicata, and 1 in Sardinia regions. Individual insects were dissected to determine nematode presence, and different nematode species were also recovered from red palm weevil cocoons collected at the sites in Apulia. Individual nematodes were molecularly identified by sequencing the ITS, D2‐D3 expansion domains of the 28SrRNA gene and the mitochondrial COI and inferring the phylogenetic relationships. The insect‐associated nematofauna identified belonged to the families Rhabditidae, Cephalobidae, and Diplogastridae. Just two nematode species, *Teratorhabditis synpapillata* and *Mononchoides macrospiculum*, were always found in association with adult insects and cocoons taken from all sampling sites. This paper reports on the biodiversity of the nematodes associated with *R. ferrugineus* and on current knowledge of the specific habitat of specialized and divergent entomophilic nematodes.

## Graphical abstract

The manuscript concerns the molecular identification of nematodes associated with *Rhynchophorus ferrugineus* by using the reverse taxonomy approach. The aim of this study was to characterize the nematofauna at level of ecological, evolutionary, and population genomics. For the first time, it has been shown that two nematode species are specifically associated with the RPW, the other species were incidentally associated to the RPW which constitute mobile microhabitat used by nematodes to feed, to move and to be transported.

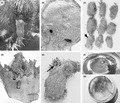

## 1 INTRODUCTION

The red palm weevil (RPW) *Rhynchophorus ferrugineus* (Olivier) (Coleoptera: Curculionidae) is considered the most damaging pest of several palm species of the family Arecaceae worldwide, especially in the Mediterranean area (EPPO, 2008a; Giblin‐Davis, Kanzaki, & Davies, 2013; Mazza et al., 2014) and is reported to infest 19 palm species. RPW originated in tropical Asia, then spread to Africa, and has recently been introduced into Europe, where it has destroyed many *Phoenix canariensis* Chabaud palms (Ferry & Gómez, 2002). RPW can occasionally attack the native dwarf fan palm, *Chamaerops humilis* (L.), and also *Washingtonia filifera* (Lind.), although these have also been reported as resistant to *R. ferrugineus* (Barranco, Pena, Martín, & Cabello, 2000; Dembilio, Jacas, & Llácer, 2009).

In Italy, RPW was reported for the first time in 2004 in Tuscany, and then spread southwards, reaching Apulia region in 2005. It was also recorded in 2005 in Sicily, where it mainly infests *P. canariensis*, but has occasionally been found also on *Washingtonia* spp., *C. humilis,* and other ornamental plants (Giovino et al., 2012). In Italy, *C. humilis* is largely cultivated along the coasts of the Tyrrhenian Sea, Sicily, and Sardinia, where most damage is caused by the South American palm borer *Paysandisia archon* (Burmeister) (Lepidoptera: Castniidae), which seems to reduce the plants' resistance to *R. ferrugineus* (EPPO, 2008b; Giovino et al., 2012).

Associations between *Rhynchophorus* species and nematodes are well documented for *R. palmarum* (L.) and *R. cruentatus* (F.), but very little is known about *R. ferrugineus* with regard to its fitness, evolution, coadaptation, speciation, defense, chemical communication, or pest management (Camerota et al., 2016; Esparza‐Diaz, Olguin, Carta, Skantar, & Villanueva, 2013; Giblin‐Davis et al., 2013; Kanzaki & Giblin‐Davis, 2018). Nematodes are microscopic worms that are adapted to living in a variety of environments, and many can be associated with other organisms that provide them with shelter or transportation, for example, entomophilic, saprobiotic, phoretic, commensal, and parasitic nematodes (Kanzaki, 2017). The number of entomophilic nematodes associated with weevils is hard to determine because nematode identification at the species level is difficult. However, it is known that every insect species can be associated with 1–5 host‐specific nematode species (Giblin‐Davis et al., 2013; Kanzaki, Giblin‐Davis, Gonzalez, & Manzoor, 2017). Two nematode species, *Teratorhabditis palmarum* Gerber & Giblin‐Davis, 1990 and *Acrostichus palmarum* Kanzaki & Giblin‐Davis, 2018 are naturally found associated with and distributed in *R. palmarum*, while the other nematode species observed, *Bursaphelenchus cocophilus* (Cobb) Baujard, *B. gerberae*, *Caenorhabditis angariae,* and *Mononchoides* sp., are occasionally carried by *R. palmarum* (Kanzaki et al., 2008; Kanzaki & Giblin‐Davis, 2018; Kanzaki, Giblin‐Davis, Zeng, Ye, & Center, 2009; Sudhaus, Kiontke, & Giblin‐Davis, 2011). *Teratorhabditis palmarum*, *Acrostichus rhynchophori* Kanzaki et al., 2009, and *Mononchoides* sp. Gerber & Giblin‐Davis, 1990 were also found sympatrically associated with *R. cruentatus*, together with other nematode species. In *R. ferrugineus*, the only reported nematode associates were *Teratorhabditis synpapillata* and *Praecocilenchus ferruginophorus* (Rao & Reddy, 1980; Kanzaki et al., 2008). However, a close association of *Mononchoides macrospiculum* and *T. synpapillata* was recently found in *R. ferrugineus* from Apulia (Southern Italy) (Troccoli, Oreste, Tarasco, Fanelli, & Luca, 2015). To date, few studies have been conducted on nematodes associated with *R. ferrugineus* to evaluate the possibility that all *Rhynchophorus* species can act as vectors of pathogenic nematodes, introducing them to new areas and new host plants.

Recent studies demonstrate that sequencing and phylogenetic analysis is a useful approach when dealing with small and/or scant specimens and taxon diversity cannot be determined with traditional approaches (Hazir, Kanzaki, Gulcu, Hazir, & Giblin‐Davis, 2015; Kanzaki et al., 2012; Markmann & Tautz, 2005). Our study used sequencing and phylogenetic approaches to investigate the biodiversity of nematodes associated with *R. ferrugineus* samples collected in southern Italy. The main goals of the present study were as follows: (a) to collect RPW adults, pupae, and cocoons from different sampling sites in southern Italy; (b) to use sequencing and phylogenetic profiles to identify all nematodes associated with RPW; (c) to compare nematode associates among different geographical sites and palm hosts; and (d) to understand the evolution and phylogeny of nematode species and of their host.

## 2 MATERIALS AND METHODS

### 2.1 Insect collection and nematode isolation

**Figure 1:**
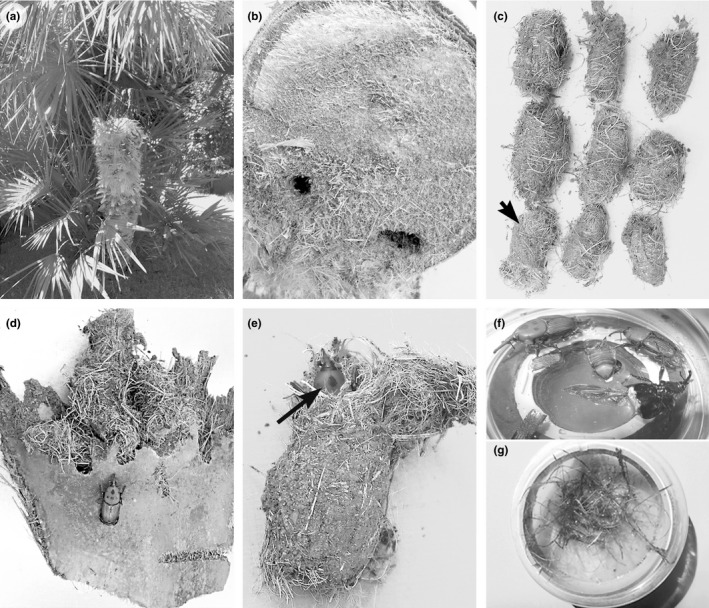
*Chamaerops humilis* palm infected by *Rhynchophorus ferrugineus* (a); symptoms of infected *C. humilis* trunk (b); cocoons of *R. ferrugineus* and *Paysandisia archon* indicated by arrow (c); basal leaf portion of *Phoenix dactilifera* containing cocoons (d); cocoon with *R. ferrugineus* (e); adult insect during dissection (f); cocoon material incubated in water for nematode collection (g)

In 2015 and 2016, RPW samples were collected from *P. canariensis* Chabaud, *P. dactylifera* L., and *C. humilis* L. palms in southern Italy presenting symptoms of RPW damage (Figure 1) 11 sites in Apulia, 1 in Sardinia, and 1 in Basilicata (Table 1). The sample unit was defined as the weevils obtained from each infested tree. Cocoons*,* pupae, and adults were collected by debarking, placed individually in plastic bags and stored in a refrigerator at 8°C. Each RPW sample was dissected under a stereomicroscope (Figure 1f), and the external surface (including elytra), hemocoel, and reproductive system were examined separately to check for the presence of nematodes (Table 1; Figure 1). The isolated specimens were observed under a stereomicroscope to determine their feeding habits.

**Table 1:** Locality and BLAST results using ITS and D2‐D3 expansion domains of nematodes associated with *Rhynchophorus ferrugineus*

Region	Site		GPS coordinates	Host plant	No. sites	No. insects	Nematode associates	GenBank Accession number		Identity %	
								ITS	D2‐D3	ITS	D2‐D3
Apulia	Bari‐Campus	(BA)	N 41.109089°	*Phoenix canariensis*	1	5	*Mononchoides macrospiculum*	LN827623	LN827616	99	100
			E 16.879650°				*Teratorhabditis synpapillata*	LN827626	AB269817	100	100
	Monopoli	(BA)	N 40.96021°	*Phoenix canariensis*	4	13	*Mononchoides macrospiculum*	LN827623	LN827616	99	100
			E 017.28381°				*Teratorhabditis synpapillata*	LN82762	AB26981	100	100
			N 40.954044°				*Oigolaimella attenuata*		KJ877276		99.72
			E 017.30872°				*Poikilolaimus* sp.	No corresponding sequences	No corresponding sequences		
			N 40.93986°								
			E 017.31256°								
	Conversano	(BA)	N 40.95015°	*Phoenix canariensis*	1	6	*Mononchoides macrospiculum*	LN827623	LN827616	99	100
			E 017.12207°				*Teratorhabditis synpapillata*	LN82762	AB26981	100	100
							Rhabditidae	No corresponding sequences	No corresponding sequences		
	Toritto	(BA)	–	*Phoenix dactiliphera*	1	8	*Mononchoides macrospiculum*	LN827623	LN827616	99	100
							*Teratorhabditis synpapillata*	LN82762	AB26981	100	100
	Ostuni	(BR)	N 40.78768°	*Phoenix canariensis*	1	4	*Mononchoides macrospiculum*	LN827623	LN827616	99	100
			E 017.58508°				*Teratorhabditis synpapillata*	LN82762	AB26981	100	100
	San Vito	(BR)	N 40.72231°	*Phoenix canariensis*	1	13	*Mononchoides macrospiculum*	LN827623	LN827616	99	100
			E 017.74842°				*Teratorhabditis sy* *npapillata*	LN82762	AB26981	100	100
							*Acrobeloides nanus*	KY828308	KX669640	98–99	100
							*Oscheius tipulae*	KJ938579	MK294532	99.6	100
	Specchiolla	(BR)	N 40.735509°	*Chamerops humilis*	1	3	*Mononchoides macrospiculum*	LN827623	LN827616	99	100
			E 17.738917°								
	Palagiano	(TA)	N 40.51116°	*Phoenix canariensis*	1	6	*Mononchoides macrospiculum*	LN827623	LN827616	99	100
			E 016.99177°				*Teratorhabditis synpapillata*	LN82762	AB26981	100	100
							*Acrostichus* sp.	LC374584		100	
Basilicata	Matera	(MT)	–	*Phoenix canariensis*	1	4	*Teratorhabditis synpapillata*	LN82762	AB26981	100	100
Sardinia	Olmedo	(SS)	–	*Phoenix canariensis*	1	9	*Teratorhabditis synpapillata*	LN82762	AB26981	100	100
							*Panagrellus* sp.		LT908055		99.6

### 2.2 DNA extraction, PCR amplification, and sequencing

Total DNA was extracted from individual nematodes obtained from dissected weevils and directly amplified as described by De Luca, Fanelli, Vito, Reyes, and Giorgi (2004). The portion of the mitochondrial cytochrome oxidase c subunit 1 (*mt*COI) gene was amplified with the primer set: COI‐F1 (5′‐CCTACTATGATTGGTGGTTTTGGTAATTG‐3′) and COI‐R2 (5′‐GTAGCAGCAGTAAAATAAGCACG‐3′) (Kanzaki & Futai, 2002); the ITS1‐5.8S‐ITS2 regions were amplified using forward primer TW81 (5′‐GTTTCCGTAGGTGAACCTGC‐3′) and reverse primer AB28 (5′‐ATATGCTTAAGTTCAGCGGGT‐3′) (Joyce, Reid, Driver, & Curran, 1994); the D2A‐D3B expansion segments of 28S rRNA gene using the primers D2A (5′‐ACAAGTACCGTGGGGAAAGTTG‐3′) and the D3B (5‐TCGGAAGGAACCAGCTACTA‐3′) (Nunn, 1992).

D2‐D3 and ITS‐RFLP analyses were performed on PCR products from individual nematodes and digested using five units of the following restriction enzymes: *Alu* I, *Ava* II, *Hinf* I, and *Rsa* I (Roche Diagnostics, Manheim, Germany). The restricted fragments were then separated on a 2.5% agarose gel by electrophoresis. The gels were stained with gel red, visualized on a UV transilluminator, and photographed using a digital system.

The D2‐D3 amplified products were purified using the protocol suggested by the manufacturer (High Pure PCR elution kit) and directly sequenced. Purified ITS and COI fragments were cloned and sent to MWG‐Eurofins in Germany for sequencing in both directions. Several specimens were not sequenced successfully.

### 2.3 Phylogenetic analysis

BLAST search at NCBI used all the new sequences obtained, which were submitted to the database and compared with the corresponding sequences to identify the closest matching nematode taxonomic and/or phylogenetic groups. Multi‐alignment was performed using the MAFFT program (Katoh & Standley, 2013). Phylogenetic trees were reconstructed with the maximum likelihood (ML) method using MEGA version 7 software (Tamura, Stecher, Peterson, Filipski, & Kumar, 2013). ML analysis under a general time‐reversible and gamma‐shaped distribution (GTR+G) model was used for D2‐D3 domains and ITS datasets. The phylograms were bootstrapped 1,000 times to assess the degree of support for the phylogenetic branching indicated by the optimal tree for each method. The trees enabled placement of all sequences not clearly assigned by Blast. The newly obtained sequences were submitted to GenBank with the following accession numbers: LR594488–LR594503 for the D2‐D3 expansion domain of the 28S rRNA gene; LR594504–LR594512 for the ITS containing region; LR594680–LR594687 for the mitochondrial COI.

## 3 RESULTS

Table 1 reports the sampling sites of palm trees with symptoms of RPW damages (Figure 1a). Nematode occurrence differed significantly among RPW specimens and across sites. All investigated cocoons, adult males and females, larvae, and pupae of *R. ferrugineus* harbored several nematode associates, most of which were dauer juveniles showing morphological similarities, which thus made species identification difficult even for specialists. However, 184 individual nematodes out of 330 were successfully amplified, and 33 high‐quality sequences were obtained (15 for the D2‐D3 expansion domains, 9 for ITS, and 8 for mitochondrial COI). PCR‐RFLP analyses were performed on ITS and D2‐D3 expansion domains to determine those specific for *M. macrospiculum* and *T. synpapillata*, avoiding sequencing of those products (data not shown). Sequences showing ≥97% similarity with those present in the database identified nematode species, while those sequences with identity thresholds <97% identified nematode sequences at the family or order level with the closest matching sequences (Floyd, Abebe, Papert, & Blaxter, 2002; Lawley, Ripley, Bridge, & Convey, 2004).

In the RPW samples from Apulia, nematodes were found under the elytra and in the hemocoel of adults, and pupae and inside the cocoons and dead palm tissues of *P. canariensis*, *P. dactylifera* and *C. humilis* (Figure 1). Two nematode species phoretically associated in the hemocoel and external surface of all RPW samples from *P. canariensis* and *P. dactylifera* were molecularly identified as *T. synpapillata* and *M. macrospiculum* by their 100% sequence identity with those in the database (Kanzaki et al., 2008; Troccoli et al., 2015), while only *M. macrospiculum* was detected in *C. humilis*. Apart from the two phoretic nematode species found in this study, other nematode species were also occasionally associated with *R. ferrugineus*. Of these, *Oscheius tipulae* (Lam & Webster, 1971), Sudhaus, 1993. *Acrobeloides nanus* (de Man, 1880), Anderson, 1968 and *Oigolaimella attenuata* (Fürst von Lieven & Sudhaus, 2008) had an identity threshold ≥97%, while *Panagrellus* sp., A*crostichus* sp., and Rhabditidae had an identity threshold <97%. The mitochondrial COI sequences had no corresponding sequences in the database and were thus identified at the family level as Rhabditidae.

One nematode *Acrostichus* sp. and several Rhabditidae were isolated from cocoons and pupae of *R. ferrugineus* collected at Palagiano (Taranto Province) (Table 1). *Acrostichus* nematodes showed a mean internal infestation of around fifteen specimens. Amplification of the ITS and mitochondrial COI of individual nematodes produced single fragments for both markers. Two ITS sequences were obtained, the 778 bp ITS showed a 97% similarity with the corresponding sequences of *A. rhynchophori* RGD193 (LC374583; 24 nucleotides different, five gaps) and *A. palmarum* RGD194–196 (LC374584, LC374585, LC374586; 19–20 nucleotides different, 0 gap) in the database and was thus identified as *Acrostichus* sp., while the 960 bp ITS was 100% identical to *M. macrospiculum*. Amplification of the *mt*DNA COI produced a fragment of approximately 700 bp that was cloned. Sequencing of two *mt*COI clones revealed that one clone had a 89% nucleotide similarity and 92% amino acid identity with *Diplogasteroides nix*; the second *mt*COI clone had a 97%–98% nucleotide similarity with several Rhabditidae in the database.

In Bari Province, all live or dead *R. ferrugineus* adults, cocoons, and pupae contained several Diplogastridae and Rhabditidae. Sequence analyses of the D2‐D3 expansion domains confirmed the presence of *M. macrospiculum* and *T. synpapillata* in all samples. One D2‐D3 sequence showed a 99% similarity (724/726 identities) with *O. attenuata* (KJ877276) in the database. The analysis of two *mt*COI sequences from two individual specimens that were recovered from the same insect containing *O. attenuata* revealed a 98% amino acid similarity with *Oigolaimella* spp., *Acrostichus* spp. and *Oscheius* spp. (123/125 identities, 124/125 positives) (data not shown). Sequence analysis of the ITS clones of another individual specimen recovered from the same insect containing *O. attenuata* found no corresponding sequences in the database, and these were assigned to the Rhabditidae. Dissection of insects and pupae collected from *Phoenix dactilifera* in Toritto (Bari Province) revealed just the co‐occurrence of *T. synpapillata* and *M. macrospiculum*, which were confirmed by sequencing and restriction profiles (not shown). Two of the three sampling sites in Brindisi Province contained *P. canariensis* and one contained *C. humilis*. Dissection of RPW cocoons, pupae, and dead adults collected from *P. canariensis* enabled the recovery of several unknown Rhabditidae, while RPW samples from *C. humilis* contained only *M. macrospiculum*. Furthermore, analysis of *C. humilis* samples revealed the presence of *P. archon* cocoons (Figure 1c). Analyses of the D2‐D3 nematode sequences from *P. canariensis* revealed one sequence that was 99% identical (0–2 nucleotides different) to the corresponding sequences of Italian *O. tipulae* strains in the database, and a second with 100% similarity to *A. nanus* and other *Acrobeloides* spp. Two different ITS sequences were also obtained in the same samples: one ITS sequence presented a 99%–100% similarity (3–11 nucleotides different) with the corresponding *O. tipulae* sequences in GenBank, while the other ITS sequence showed a 96%–98% similarity (753/782 and 766/784 identities, respectively, and 6–7 gaps) with *A. nanus* sequences present in GenBank. The *mt*COI sequence showed a 94% similarity with *O. tipulae* and *Oscheius* spp. present in the database (116/124 and 116/123 identities, 120/124 and 119/123 positives, respectively).

Dead RPW adults collected in Matera Province harbored 2–100 specimens belonging to *T. synpapillata* inside the hemocoel and under elytra, as confirmed by RFLP analyses of D2‐D3 and ITS products and by ITS region sequencing.

Live and dead *R. ferrugineus* samples from Sardinia harbored *T. synpapillata*, inside the hemocoel and under elytra, and also several rhabditids. The sequence of the D2‐D3 expansion domains of the rhabditids showed a 100% similarity with *Panagrellus* sp. recovered from *R. ferrugineus* in Tuscany, and from decaying pomegranate in association with the nematode *Sheraphelenchus sucus* in Apulia (Camerota et al., 2016; Fanelli et al., 2017).

### 3.1 Phylogenetic analyses

**Figure 2:**
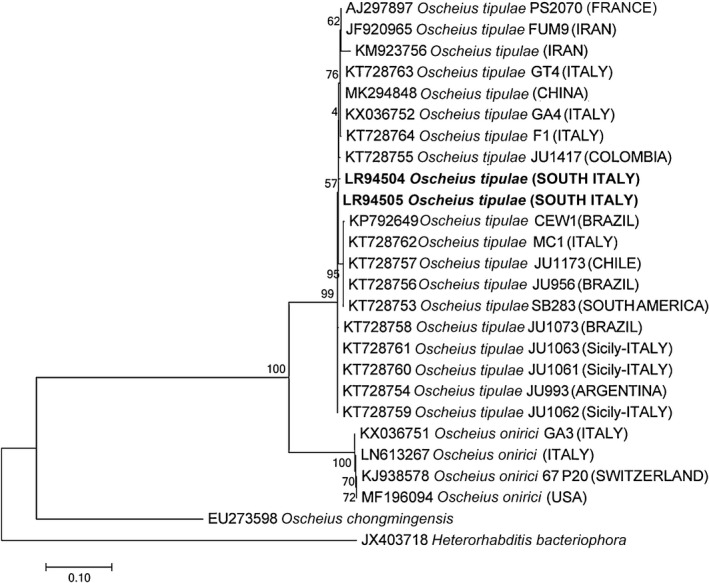
Phylogenetic tree of ITS containing region describing the evolutionary relationships among different geographical populations of *Oscheius tipulae* using maximum likelihood (ML) method. Branch lengths are proportional to the distances as derived from the distance matrix obtained using the GTR method with the invariant site plus gamma options. Numbers at nodes indicate bootstrap values

**Figure 3:**
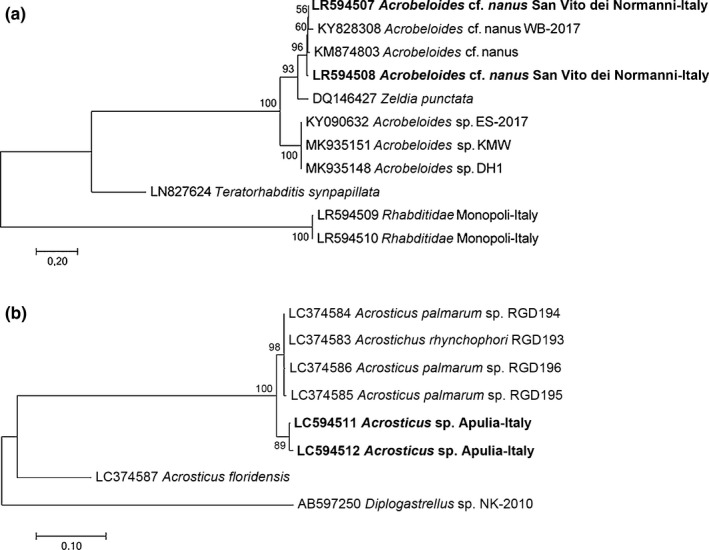
Phylogenetic tree of ITS containing region describing the evolutionary relationships of *Acrobeloides cf. nanus* (a) and *Acrostichus* sp. (b) associated with *Rhynchophorus ferrugineus* using maximum likelihood (ML) method. Branch lengths are proportional to the distances as derived from the distance matrix obtained using the GTR method with the invariant site plus gamma options. Numbers at nodes indicate bootstrap values

The newly obtained sequences of ITS and D2‐D3 expansion segments of 28S rDNA, were multi‐aligned with the closest sequences in GenBank and ML was used to reconstruct the phylogenetic trees (Figures 2 and 3). The ITS tree confirmed the clustering of *O. tipulae* (100% support) with the corresponding *O. tipulae* database sequences from different geographical origins, in Italy, South America and Europe (Figure 2), and of *A. nanus* (100% support) with the corresponding database sequences (Figure 3a), while the *Acrostichus* sp. sequences obtained in this study showed sister relationships with *A. palmarum* and *A. rhynchophori* sequences (Figure 3b). The phylogenetic tree of the D2‐D3 expansion segments of 28S rDNA (data not shown) grouped the new sequences with the corresponding sequences from the database. The *mt*COI phylogenetic tree revealed the presence of nematode species not yet present in the database, and so no phylogenetic tree is presented.

## 4 DISCUSSION

The present study confirmed the co‐occurrence of two phoretic nematode species, *T. synpapillata* and *M. macrospiculum*, associated with RPW at all sampling sites (Troccoli et al., 2015) and provided information regarding other nematodes potentially associated with RPW in southern Italy. Individual insects can simultaneously harbor more than one nematode genus (Grucmanová, Holuša, Čermák, & Nermut, 2015; Kanzaki et al., 2011; Shimizu et al., 2013); *R. palmarum* and *R. cruentatus* are reported to host several nematode associates, including *Teratorhabditis* and *Mononchoides* spp. (Esparza‐Diaz et al., 2013; Kanzaki et al., 2008, 2017, 2009; Mazza et al., 2014; Sudhaus et al., 2011). This finding confirmed the possibility of vertical transmission between *Teratorhabditis* and *Mononchoides* nematode genera and the insect genus *Rhynchophorus* (Kanzaki et al., 2008; Troccoli et al., 2015), suggesting that the ecological niche plays an important role in shaping the nematode community of *Rhynchophorus* spp. Furthermore, this result suggests an important role for both nematode genera in supporting RPW during palm tree attacks. Similarly, it has been demonstrated very recently that insect‐associated nematodes in the dung beetle *Onthophagus taurus* (Schreber) positively influence the development of its offspring, thus contributing to the wealth of insect species (Ledón‐Rettig, Moczek, & Ragsdale, 2018). The co‐occurrence of RPW and *P. archon* in *C. humilis* confirmed that the palm's antixenotic mechanism can be by‐passed when it is attacked by *P. archon* (EPPO, 2008b). When sequence data of other nematode species isolated from RPW were compared with those in GenBank and those isolated from other palm weevils, they were identified as *O. attenuata*, *A. nanus*, *O. tipulae*, *Acrostichus* sp., and *Panagrellus* sp. together with several unknown Rhabditidae. Three nematode species *O. tipulae*, *O. attenuata,* and *A. nanus* were recorded here for the first time in association with *R. ferrugineus*, whereas *Panagrellus* sp. had already been reported in association with *R. ferrugineus* in Tuscany, and *Acrostichus* sp. with other congeneric weevil species, that is, *R. palmarum and R. cruentatus* (Félix, 2006; Kanzaki & Giblin‐Davis, 2018).

Two other nematode species, *O. tipulae* and *A. nanus*, were isolated sympatrically from the same cocoons containing dead pupae from a site in Brindisi Province, suggesting that these nematodes share the same habitat and do not compete for feeding sources. The natural association of *O. tipulae* with *Acrobeloides* spp. on insect cadavers has already been reported (Campos‐Herrera et al., 2015). The phylogenetic trees using ITS and D2‐D3 sequences of the Apulian *O. tipulae* strain grouped the Italian *O. tipulae* sequences together with the corresponding sequences in the database (100% support) and showed sister relationships with *Oscheius onirici* (Figure 2). Furthermore, our study confirmed the hypothesis of Torrini et al. (2016) that all Italian *O. tipulae* strains could have been introduced from South America in association with plants or insects. Our results clearly prove that Italian *O. tipulae* strains can be isolated from soil and rotting fruits or found in association with *R. ferrugineus*. This nematode species was firstly reported to be associated with larvae of the tipulid dipteran *Tipula paludosa* Meigen (Lam & Webster, 1971) and was recently recovered in Iran in association with bark samples containing bark beetle galleries (Valizadeh, Goldasteh, Rafiei‐Karahroodi, & Pedram, 2017). More recently, Karimi, Rezaei, and Shokoohi (2018) have also recovered *O. tipulae* from soil in Iran using *Galleria* bait, thus suggesting *O. tipulae* as a potential entomopathogenic nematode.

With regard to the *A. nanus* population we found in Apulia, we recorded the first occurrence in *R. ferrugineus* of this nematode, which has previously been reported only in earthworm cocoons (Kraglund & Ekelund, 2002). Campos‐Herrera et al. (2015) recently observed the co‐occurrence of free‐living nematodes of the *Acrobeloides* and *Oscheius* genera on insect cadavers used as a food source. Our results demonstrate the co‐occurrence of *A. nanus* and *O. tipulae* in an individual *R. ferrugineus* cocoon, confirming that both nematode species can share the same carrier and the same habitat without evident interaction.

The *Acrostichus* sp. found in the RPW samples we collected in Apulia was phylogenetically close to *A. palmarum* and *A. rhynchophori* (100% support) (Figure 3b). Since the *Acrostichus* sp. was not broadly associated with Apulian RPWs, the nematode was found in only a few insects from just one sampling site (Palagiano‐Taranto Province), thus suggesting that this population is native to Apulia. Kanzaki and Giblin‐Davis (2018) have recently demonstrated that *A. palmarum* isolated from *R. palmarum* and *A. rhynchophori* isolated from *R. cruentatus* are cryptic species, associated with different species of *Rhynchophorus* occupying different geographical areas. Thus, our finding supports the observation that host switching to related weevils causes parallel divergence or cospeciation of these nematodes. This is also corroborated by the results obtained for bee‐associated *Acrostichus* spp., suggesting vertical or sexual transmission to explain the evolution of host specificity and cophylogeny (McFrederick & Taylor, 2012).

Dissection of cocoons from Monopoli (Bari Province) revealed *T. synpapillata* and *M. macrospiculum*, in addition to the co‐occurrence of two other nematode species, which were molecularly identified as *O. attenuata* unknown Rhabditidae. The copresence of the omnivore *O. attenuata* with bacterial feeders has already been reported in wood‐boring insects (Fürst von Lieven & Sudhaus, 2008; Kanzaki, 2017).

Surprisingly, the bacterial feeder nematode *Panagrellus* sp. was recovered from the hemocoel of several *R. ferrugineus* specimens from Olmedo (Sardinia). This is a second occurrence of *Panagrellus* sp. from *R. ferrugineus* in Italy; but, it was also found to co‐occur in Italy with the mycetophagus nematode *S. sucus* in rotting and decaying pomegranates, with a likely association with *Drosophila* fruit flies. These observations confirm that the Italian *Panagrellus* sp. has an entomophilic relationship with different insects and that host switching occurs when it occasionally occupies the same habitat as *R. ferrugineus*.

The association patterns observed in our study suggest that the nematode species associated with a few Italian RPW may be incidentally associated, or else subject to host switching because nematodes and insects share similar environmental conditions. Furthermore, the low number of specimens recovered from each insect can be explained by competition for food sources, seasonal environmental changes, and competition with other microbes sharing the same palm habitat and insect host.

The presence of different nematodes associated with *R. ferrugineus* confirms the low association rate between nematodes and RPW, suggesting that RPW is probably not the typical or primary host for these nematodes.

In conclusion, the molecular approach allows us to assign anonymous sequences to *taxon* groups representing different trophic levels, and to determine *taxon* diversity in the context of ecological analysis. The present study demonstrates that most nematode associates are morphologically similar Rhabditidae and are thus difficult to identify at the species level. Furthermore, this study reveals a specific association of *T. synpapillata* and *M. macrospiculum* with RPW in southern Italy. Other nematode associations with the native RPW can occur incidentally because these new nematode associates share the same habitat as RPW, which acts as an occasional carrier. Although the origins of these associations are in most cases not clear, it appears that an important role in the evolution of these interesting entomophilic nematodes is played by associations with soil or possibly with other moist habitats, followed by host‐carrier switching.

## CONFLICT OF INTEREST

The authors declare no conflict of interest.

## AUTHOR CONTRIBUTIONS

F.D.L. designed the study, analyzed the data, and wrote the paper; E.F designed the study and performed the experiments; M.O., G.S., A.T., and E.T. conducted insect dissection and morphological identification; A.V. and N.T conducted palm surveys.

## Data Availability

RFLP profiles of ITS and D2‐D3 expansion segments of several populations of *Teratorhabditis synpapillata* and *Mononchoides macrospiculum* from southern Italy and the phylogenetic tree using D2‐D3 sequences are available at Dryad data repository: https://doi.org/10.5061/dryad.ffbg79cqg
